# Nephroprotective Role of Resveratrol and Ursolic Acid in Aristolochic Acid Intoxicated Zebrafish

**DOI:** 10.3390/toxins7010097

**Published:** 2015-01-13

**Authors:** Yu-Ju Ding, Chiao-Yin Sun, Chi-Chung Wen, Yau-Hung Chen

**Affiliations:** 1 Department of Chemistry, Tamkang University, No. 151 Ying-chuan Road, Tamsui, New Taipei City 25137, Taiwan; E-Mail: dingfion@gmail.com; 2 Department of Nephrology, Chang Gung Memorial Hospital, Keelung 20491, Taiwan; E-Mail: fish3970@gmail.com; 3 Department of Mathematics, Tamkang University, Tamsui, New Taipei City 25137, Taiwan; E-Mail: ccwen@mail.tku.edu.tw

**Keywords:** aristolochic acid, kidney, nephrotoxicity, resveratrol, ursolic acid, zebrafish

## Abstract

The nephrotoxicity of aristolochic acid (AA) is well known, but information regarding the attenuation of AA-induced toxicity is limited. The aim of the present study was to study the nephroprotective effects of resveratrol (Resv) and ursolic acid (UA) in a zebrafish model. We used two transgenic lines, *Tg(wt1b:EGFP)* and *Tg(gata1:DsRed)*, to evaluate the nephroprotective effects of Resv and UA by recording subtle changes in the kidney and red blood cell circulation. Our results demonstrated that both Resv and UA treatment can attenuate AA-induced kidney malformations and improve blood circulation. Glomerular filtration rate assays revealed that both Resv and UA treatment can restore renal function (100% for Mock; 56.1% ± 17.3% for AA-treated; 80.2% ± 11.3% for Resv+AA; and 83.1% ± 8.1% for UA+AA, *n* = 15). Furthermore, real-time RT-PCR experiments showed that pre-treatment with either Resv or UA suppresses expression of pro-inflammatory genes. In conclusion, our findings reveal that AA-induced nephrotoxicities can be attenuated by pre-treatment with either Resv or UA. Therefore, we believe that zebrafish represent an efficient model for screening AA-protective natural compounds.

## 1. Introduction

Serious damage to the kidney, a very delicate organ, increases the rate of uremic bleeding. This leads to renal failure, a life-threatening disease. Most of the patients undergo dialysis as a long-term treatment because no specific medication has been developed [1]. Therefore, investigation of the causes of renal damage and development of new methods for preventing renal damage are needed.

There are many risk factors that have been demonstrated to be highly associated with renal failure. Exposure to xenobiotics, such as antibiotics, heavy metals, aristolochic acid-containing traditional Chinese herbs and other natural toxins, is considered to be the main cause of renal damage [2,3]. Accompanied by xenobiotic-induced inflammation, such exposures often cause serious kidney injuries. For example, mice treated with cisplatin had an accumulation of leukocytes and upregulation of pro-inflammatory molecules, which consequently caused tubular damage and acute kidney injury [4]. These observations indicate that exposure to a toxin, combined with subsequent inflammation, increase the risk of renal damage.

Aristolochic acid (AA) is found primarily in herbal extracts and can induce clinical nephropathy [5,6]. Epidemiological studies have shown that aristolochic acid nephropathy (AAN) is associated with a high long-term risk for renal failure and urothelial cancer [7,8]. In Taiwan, approximately one-third of the population has been prescribed herbal remedies containing *Aristolochia*, and the recorded incidence of urinary tract cancers is the highest in the world [9]. In several AAN patients, foci of inflammatory cells infiltrating into the corticomedullary junctions were also found [10]. In rats, accumulation of monocytes/macrophages and T lymphocytes and AA-induced inflammation cause renal injury [11]. In zebrafish, our previous study showed that AA-exposure caused acute inflammation that led to malformation of embryonic zebrafish heart and kidney [12].

Because drug-induced inflammation may be the major cause of renal failure, anti-inflammatory agents should have the potential to attenuate renal failure. Resveratrol (Resv) and ursolic acid (UA) are rich in grape and apple peels, both of which are well-known natural anti-inflammatory agents [13,14]. It has been shown that UA can be used to prevent liver damage through restraining the activation of toxic ingredients, and boosting immunity [15,16,17]. Moreover, Resv is found to be capable of simultaneously restraining the production of reactive oxygen species (ROS) within and without cells. As a result, these agents can prevent diseases caused by oxidative stress. Castro* et al.* [18] reported that Resv can reduce the expression of MPO and restrain the production of inflammation. Because of their anti-inflammatory activity, Resv and UA are often used to prevent and treat immune-mediated diseases [19,20]. These observations suggest that Resv and UA may have the potential to attenuate or to prevent inflammation-induced renal malformations.

Zebrafish represent a very effective animal model for toxicological studies [21,22,23,24,25,26]. In the present study, we attempted to investigate the nephroprotective roles of Resv and UA in a zebrafish model. We utilized the transgenic lines *Tg(wt1b:EGFP)* and *Tg(gata1:DsRed)* to study the resulting subtle changes in the kidney and the red blood cells, respectively. We also generated a series of time- and dose-dependent AA exposure experiments. We hope to establish a whole-organism drug screening platform to select more useful natural compounds for the prevention of nephropathy.

## 2. Results and Discussion

### 2.1. Resv/UA Has No Evident Effects for Enhancing Survival Rates of AA-Intoxicated Zebrafish Embryos

**Figure 1:**
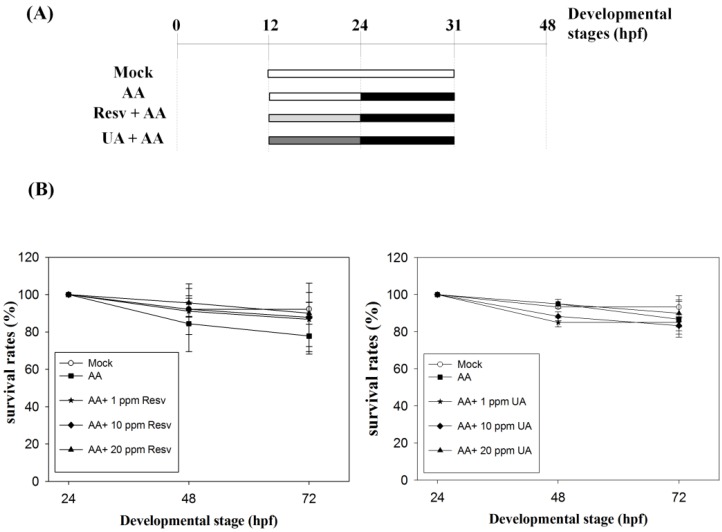
(**A**) Administration methods for the four processing modes utilized in this study. Mock: Embryos were incubated in egg water; aristolochic acid (AA): Embryos were exposed to 3 ppm AA at 24–31 hpf; and resveratrol (Resv)+AA or ursolic acid (UA)+AA groups: Embryos were treated with Resv or UA at 12–24 hpf (1, 10 and 20 ppm) and then exposed to AA at 24–31 hpf; (**B**) Survival rates of embryos in the prevention method. Embryos were incubated in water (mock), exposed to 3 ppm AA only (AA control), or treated with 1, 10 and 20 ppm Resv or UA following exposure to 3 ppm AA. The *X*- and *Y*-axes represent the developmental stages and the survival rates, respectively.

To study the protective effects of Resv/UA on AA-induced renal malformations, we first treated zebrafish embryos with AA (3 ppm) at 24–31 hpf to cause kidney malformation and then treated with the desired concentrations (0, 1, 10 and 20 ppm) of either Resv or UA and calculated their survival rates (Figure 1). The results revealed that 77.8% ± 9.6% to 86.7% ± 9.6% (mean ± standard error; *n* = 30 (number of embryos; *N* = 5, repeated for 5 times) of the AA-exposed embryos were alive at 72 hpf, while 83.3% ± 4.7% to 90.0% ± 9.4% (*n* = 30, *N* = 5; UA+AA) and 86.7% ± 14.5% to 90.0% ± 5.8% (*n* = 30, *N* = 5; Resv+AA) of embryos were alive, indicating no evident differences in survival rates between AA treatment and prevention groups (UA+AA and Resv+AA).

### 2.2. Resv and UA Can Attenuate Aristolochic Acid-Induced Kidney Malformation

**Figure 2:**
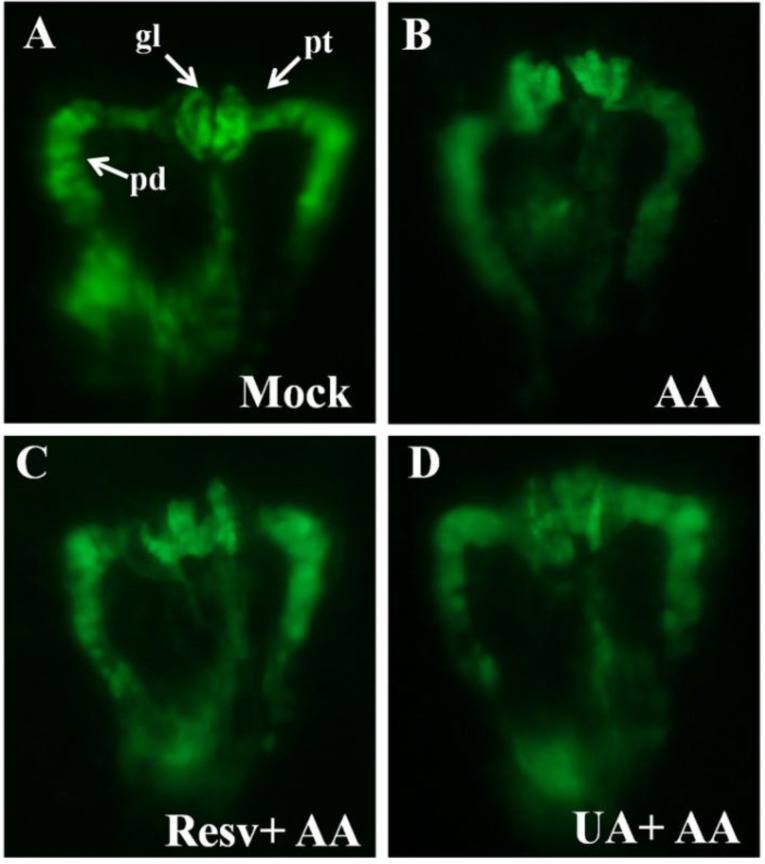

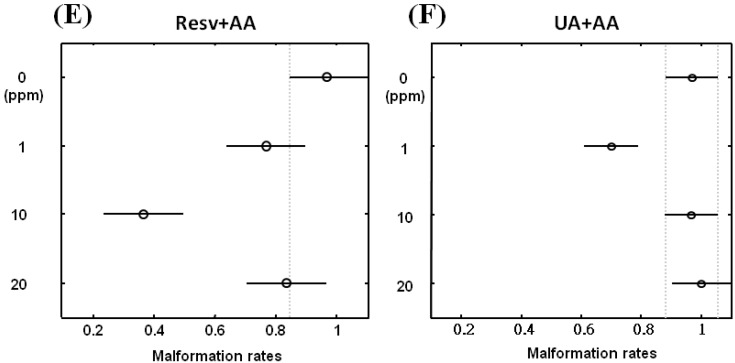
Kidney phenotypes of zebrafish embryos after prevention (10 ppm Resv or UA). (**A**) No defect of mock group; (**B**) AA caused atrophic glomerulus and pronephric tube (pt) curved defect; (**C** and **D**) Prevention groups treated to restore a similar phenotype as the mock group. All kidney photos were taken from the dorsal view at the developmental stage of 48 hpf. *Tg(wt1b:EGFP)*. pt, pronephric tubule; pd, pronephric duct; gl, glomerulus; (**E**,**F**) The Tukey-Kramer HSD (honestly significant difference) test: the mean malformation rates and their 95% confidence intervals for treatment groups. Two group means were considered significantly different if their intervals were disjointed and were not significantly different if their intervals overlapped.

The transgenic *Tg(wt1b:EGFP)* zebrafish, in which the glomerulus, pronephric tube, pronephric duct and exocrine pancreas can be easily observed by green fluorescence, is a useful tool for studying nephrotoxicities, especially for high throughput pipeline analysis [12,25,26]. The present study employed the transgenic line *Tg(wt1b:EGFP)* as a model to evaluate the protection effects of Resv and UA. Our results showed that AA-exposed zebrafish displayed more malformed kidney phenotypes than those in the mock control group at 48 hpf. Those malformed kidney phenotypes include (1) curved and dilated pronephric tubules and (2) a swollen and unfused glomerulus (Figure 2A* vs.* 2B). Interestingly, those AA-induced kidney malformations can be attenuated by pre-treatment with either Resv or UA (Figure 2C,D).

To further evaluate the protective effects of Resv and UA using statistical analyses, we first applied the ANOVA method to examine the dosage effect (0, 1, 10 and 20 ppm) on the malformation rate for the two drugs (Resv and UA) separately. The tests give very small *p-*values (<0.0001) for both “Resv” and “UA” treatments, indicating a highly significant difference between dosage groups for each drug treatment. The Tukey-Kramer HSD test was then used for pairwise comparison of the mean malformation rates of dosage groups for the drugs “Resv” and “UA”. For “Resv”, the test reports that the mean malformation rates (standard error, sample size) for four dose groups (AA only, AA+Resv 1 ppm, AA+Resv 10 ppm, and AA+Resv 20 ppm) are 96.88% (6.80%, *n* = 32), 76.67% (7.02%, *n* = 30), 36.67% (7.02%, *n* = 30), and 83.33% (7.02%, *n* = 30), respectively. For “UA”, the mean malformation rates (standard error, sample size) for the four dose groups (AA only, AA+UA 1 ppm, AA+UA 10 ppm, and AA+UA 20 ppm) were 96.88% (4.75%, *n* = 32), 70.00% (4.91%, *n* = 30), 96.67% (4.91%, *n* = 30), and 100% (5.27%, *n* = 30), respectively. Figure 2E,F presents the mean malformation rates with 95% confidence intervals of dosage groups for drugs “Resv” and “UA”, respectively. The findings show that the malformation rates for the “AA+Resv 10 ppm” group and “AA+UA 1 ppm” group were significantly lower than that for the “AA only” group. This suggests that both Resv with a dosage of 10 ppm and UA with a dosage of 1 ppm have significant resistance effects against “AA” poisoning.

### 2.3. Resv and UA Treatment Attenuate AA-Induced Renal Failure

**Figure 3:**
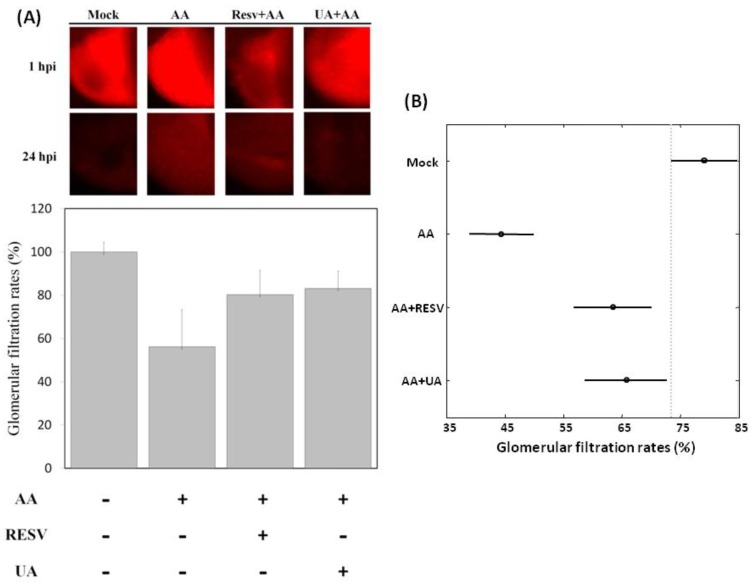
Glomerular filtration rates of Resv and UA prevent aristolochic acid nephropathy (AAN). (**A**) Zebrafish embryos were either exposed to water (no treatment) or water containing 5 ppm AA and 10 ppm Resv or UA exposure. Dextran tetramethylrhodamine was injected into the cardiac venous sinus of 72 hpf embryos derived from different groups (mock, prevention), and fluorescence intensities were recorded after 1 h post-injection (hpi) and 24 hpi (*n* = 15); (**B**) Tukey-Kramer HSD test: the mean glomerular filtration rates and their 95% confidence intervals for treatment groups (“Mock”, “AA “, “AA+Resv”, and “AA+UA”). Two group means were considered to be significantly different if their intervals were disjointed and were not significantly different if their intervals overlapped.

For further confirmation of the protective effects of Resv/UA, we performed glomerular filtration rate (GFR) assays on zebrafish embryos. As shown in Figure 3A, GFR at 72 hpf in the AA-treated group was 56.1% ±17.3% (*n* = 15) and increased to 80.2% ± 11.3% and 83.1% ± 8.1% in the Resv+AA and UA+AA groups (*n* = 15), respectively. This indicates that Resv and UA can partially restore kidney function. The average GFR (standard error, sample size) for the “mock”, “AA”, “AA+RESV”, and “AA+UA” groups were 79.15% (1.16%, *n* = 15), 44.44% (4.32%, *n* = 16), 63.50% (3.41%, *n* = 11), and 65.78% (2.55%, *n* = 10), respectively. The ANOVA test gives a very small *p-*value (<0.0001), indicating a highly significant difference in glomerular filtration rates between treatment groups. The Tukey-Kramer HSD test was then used to conduct pairwise comparisons of the average glomerular filtration rates of the different treatment groups. Figure 3B presents the average glomerular filtration rates of the treatment groups with 95% confidence intervals. The conclusions are as follows: (i) the average GFR of the “AA”, “AA+RESV”, and “AA+UA” groups were significantly lower than that of the “mock” group; (ii) the average GFR of the “AA+RESV” and “AA+UA” groups were significantly higher than that of the “AA” group; and (iii) the average GFR of the “AA+UA” group was higher than that of the “AA+RESV” group, although the difference was not significant. This suggests that Resv and UA treatment attenuate AA-induced renal failure.

### 2.4. Resv and UA Improve the Accumulation of Erythrocytes and Restoration of Blood Circulation

**Figure 4:**
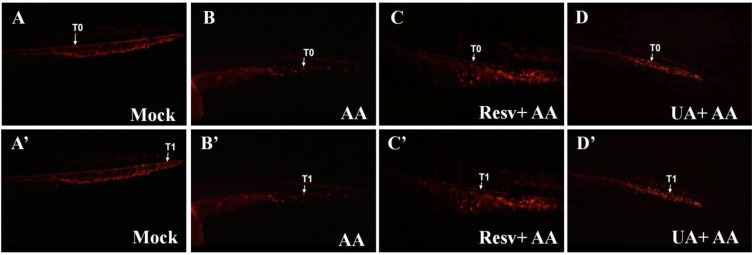
Effects of Resv or UA on blood circulation. Images were captured by live video (Supplementary movie S1) of *Tg(gata1:DsRed)* embryos without AA treatment (**A**,**A'**) after 3 ppm AA treatment (**B**,**B'**) and Resv or UA prevention groups (**C**,**C'**,**D**,**D'**). T0: Initial position of one red blood cell; T1: Position of the red blood cell 1 s later.

Transgenic *Tg(gata1:DsRed)* zebrafish, in which the red blood cells can be identified by red fluorescence, are a useful tool for studying defects in blood circulation [27]. We have previously shown that AA-treated *Tg(gata1:DsRed)* embryos had abnormal blood circulation and red blood cell accumulation in the kidney [12]. In this study, the same strategy is applied to evaluate the nephro-preventive effects of Resv and UA. As shown in the supplementary movie, the distance between T0 (initial position of one red blood cell) and T1 (position of the red blood cell 1 s later) in AA-exposed embryos was shorter than that in the mock control group, indicating that blood circulation was blocked by AA exposure (Figure 4A,A'* vs.* 4B,B'). However, the distance between T0 and T1 in the Resv or UA prevention groups was longer than that of embryos in the AA-exposed group, suggesting that blood circulation was restored after treatment with either Resv or UA (Figure 4C,C'* vs.* 4D,D').

**Figure 5:**
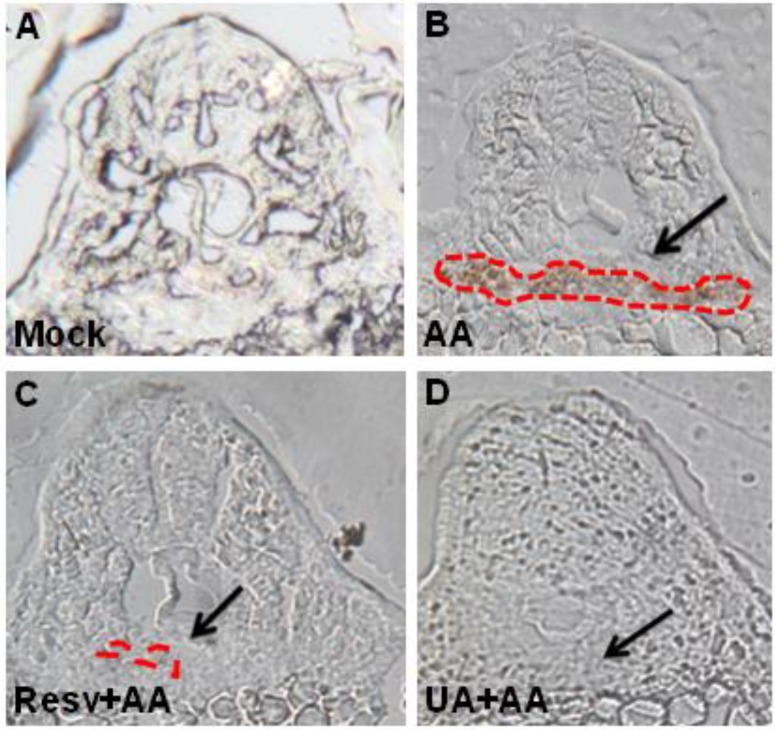
Resv or UA decrease AA-induced red blood cell accumulation. Embryos derived from the mock (no treatment: **A**), AA-treated group (**B**), and Resv (**C**) or UA prevention groups (**D**) were stained with ο-dianisidine. The black arrow indicates the position of the glomerulus. The red dots indicate the region of blood accumulation.

Moreover, as evidenced by ο-dianisidine staining, AA-exposed embryos had abnormal accumulation of red blood cells in the tubular and glomerular regions (Figure 5A* vs.* 5B), but the blood accumulation was attenuated by treatment with either Resv or UA (Figure 5C,D).

### 2.5. Treatment with Either Resv or UA Suppresses the Generation of ROS and the Inflammatory Response

**Figure 6:**
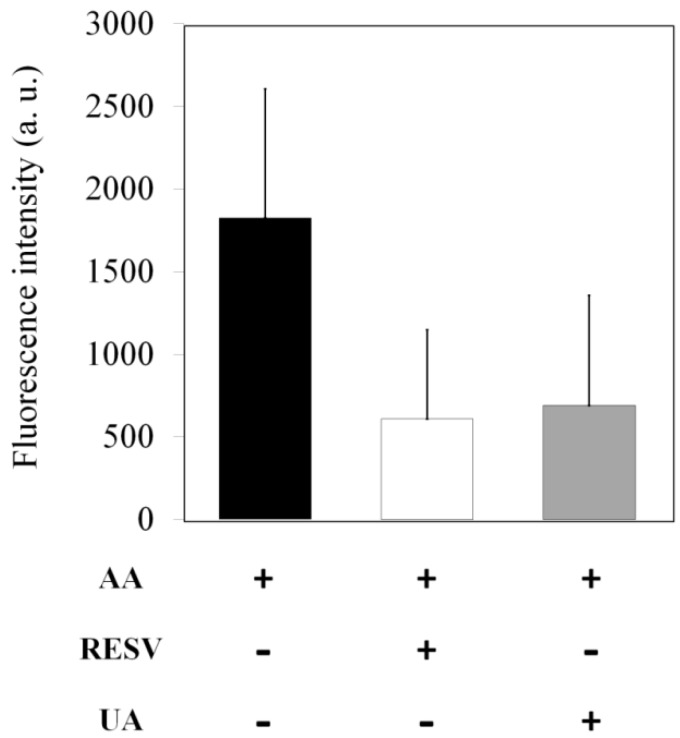
Concentration of reactive oxygen species (ROS) in zebrafish exposed to 3 ppm of AA and prevention groups (containing Resv or UA). Data (fluorescence intensity) are expressed as the mean ± standard error (*n* = 30).

It has been reported that AA treatment leads to kidney inflammation [12,28]. In this regard, we further investigated whether AA-induced reactive oxygen species (ROS) are eliminated by Resv/UA treatment. As shown in Figure 6, AA-induced ROS fluorescence intensity was 1826 arbitrary units (a.u.). In contrast, AA-induced ROS fluorescence intensity was decreased to 609 and 690 a.u. (approximately a 63%–67% reduction) after treatment with either Resv or UA, respectively. These observations suggest that both Resv and UA were able to significantly reduce ROS production in zebrafish embryos.

**Figure 7:**
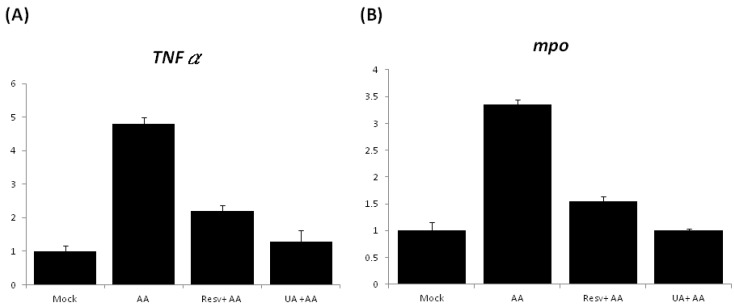
Quantitative real-time polymerase chain reaction (qPCR) analysis of the expression levels of marker genes ((**A**): *TNFα*; (**B**): *mpo*) between mock (no treatment), AA-treated, and Resv or UA prevention embryo groups.

The expression of pro-inflammatory genes was further examined by quantitative PCR (qPCR), and the results showed that the expression of *TNFα* and *mpo* in the AA-treated embryos increased by 4.8- and 3.4-fold, respectively, in comparison with the mock control embryos (Figure 7). However, the expression of *TNFα* in the Resv+AA and UA+AA groups was decreased to 2.2- and 1.3-fold (Figure 7A); expression of *mpo* in the Resv+AA and UA+AA groups was decreased by 1.5- and 1.0-fold, respectively (Figure 7B). These results suggest that the attenuation of AA-induced kidney malformation by both Resv and UA treatment may be mediated by suppression of pro-inflammatory gene expression.

## 3. Experimental Section

### 3.1. Fish Maintenance and Embryo Collection

Zebrafish (wild type, WT; AB strain), *Tg(wt1b:EGFP)* and *Tg(gata1:DsRed)* [25,26,29] were maintained in 16–20 gallon tanks at 28.5 °C under a 14/10 h light-dark cycle. Their embryos were collected according to standard criteria (hours post-fertilization, hpf, or days post-fertilization, dpf [30]. All animal experiments in this study were performed in accordance with the guidelines issued by the animal ethics committee of Tamkang University.

### 3.2. Chemical Exposure and Survival Rate Analysis

We employed a previously described aristolochic acid (AA) exposure protocol [12] to evaluate the nephroprotective effects of resveratrol (Resv; trans-3,4,5-trihydroxystilbene) and ursolic acids (UA; C_30_H_48_O_3_). In the present study, four exposure protocols were used: (I) Mock: water only; (II) AA: embryos were exposed to AA from 24 to 31 hpf; (III) Resv+AA: embryos were soaked in Resv (12–24 hpf) prior to AA exposure; and (IV) UA+AA: embryos were soaked in UA (12–24 hpf) prior to AA exposure. For dose titration and survival rate analysis, *Tg(wt1b:EGFP)* embryos (by 12 hpf) were collected (30 embryos per group) and were exposed according to the above-mentioned protocols, differing in the addition of either Resv or UA at different concentrations (0, 1, 10 and 20 ppm). All embryos were cultivated in 24-well cell culture plates, and survival rates were counted at the check point.

### 3.3. Detection of ROS and Data Analysis

To detect the accumulation of ROS, embryos were incubated with 500 ng/mL dihydrodichlorofluorescein diacetate (H_2_DCFDA). Intracellular H_2_DCFDA was de-esterified to dichlorodihydrofluorescein, which was oxidized by ROS to produce the fluorescent compound dichlorofluorescein (DCF). After a 60-min incubation at 28 °C, the fluorescence intensity (FI) of the embryos was measured at an excitation/emission = 485/530 nm.

### 3.4. Zebrafish Renal Function Assay

The procedure used for the detection of acute renal failure was described previously [31]. In brief, zebrafish embryos were anesthetized in 40 μg/mL tricaine (ethyl-m-aminobenzoate methanesulfonate salt, Sigma, St. Louis, MO, USA) and positioned in an injection holder. Tetramethylrhodamine-labeled 10-kDa dextran (Molecular Probes, Eugene, OR, USA) was injected into the cardiac venous sinus at 72 h post-fertilization, and individual fluorescence intensity at baseline, 1 and 24 h was measured in each fish. The fluorescence intensity ratios were defined using image J analysis software (1.47q, NIH, Bethesda, MD, USA, 2013).

### 3.5. Red Blood Cell Staining

The procedure for detecting erythropoiesis was described previously [32] and was used in the present study with minor modification. Embryos at 48 hpf were bathed in a stain buffer with combinations of 0.6 mg/mL *ο*-dianisidine in 40% ethanol, 10 mM sodium acetate, pH 5.2, and 0.65% hydrogen peroxide and incubated in the dark at room temperature for 15 min. Embryos were washed with PBS, fixed in 4% paraformaldehyde, and observed under a dissection microscope (Leica, Wetzlar, Germany).

### 3.6. Histology and Quantitative Reverse Transcription-Polymerase Chain Reaction (qRT-PCR)

The experimental procedures for cryosectioning and qRT-PCR have been previously described [33,34,35], except that primer sets were synthesized for detecting the expression levels of *TNFα* and *mpo*. The housekeeping gene *β-actin* was used as an internal control for normalization in quantification.

### 3.7. Images

All of the embryos were observed under a microscope (DM 2500, Leica, Wetzlar, Germany) equipped with Nomarski distinguishing interference contrast optics and a fluorescent module including GFP, DsRed and DAPI filter cubes (Kramer Scientific, Amesbury, MA, USA). Pictures of the embryos were captured at particular stages with a CoolSNAP CCD (Photometrics, Tucson, AZ, USA). A 15-s movie of the embryos at 48 hpf was recorded (SONY, Tokyo, Japan).

### 3.8. Statistical Analysis

All analyses in this study were carried out using Matlab software (version 7.7 R2008b, MathWorks, Natick, MA, USA, 2008). ANOVA (analysis of variance) was applied to examine the effect of dose on the mean malformation rate. The p-value reported by ANOVA indicated that samples in all dosage groups are drawn from the same population. The Tukey-Kramer HSD (honestly significant difference) test was further used to compare the population mean malformation rate for each dosage group. A significance level of 0.05 was used in all statistical analyses, and a family-wise error rate of 0.05 was used in the Tukey-Kramer HSD test.

## 4. Conclusions

In mice and humans, pro-inflammatory responses of renal endothelial cells and activation of inflammatory leukocytes reduce renal blood flow by vascular congestion and consequently cause kidney malformations [36,37]. Similarly, aristolochic acid (AA)-induced nephropathy is mediated by inflammation in both mice and zebrafish [12,28]. In the present study, we tested whether AA-induced nephropathy can be attenuated by treating the developing zebrafish embryos with natural anti-inflammatory agents such as resveratrol (Resv) and ursolic acid (UA). Our studies revealed that AA can induce an inflammatory response associated with the progression of circulation and kidney defects in zebrafish embryos and that such an inflammatory response can be attenuated by pre-treatment with either Resv or UA. In conclusion, we believe that zebrafish represent an efficient model for screening AA-protective natural compounds.

## Supplementary Materials

Supplementary materials can be accessed at: http://www.mdpi.com/2072-6651/7/1/0097/s1.
